# Determinants of Willingness for COVID-19 Vaccine: Implications for Enhancing the Proportion of Vaccination Among Indians

**DOI:** 10.7759/cureus.15271

**Published:** 2021-05-27

**Authors:** Jaison Jacob, Shine Stephen, Alwin Issac, Nadiya Krishnan, Rakesh Vadakkethil Radhakrishnan, Vijay V R, Manju Dhandapani, Sam Jose, Azhar SM, Anoop S Nair

**Affiliations:** 1 College of Nursing, All India Institute of Medical Sciences, Bhubaneswar, IND; 2 Psychiatry, All India Institute of Medical Sciences, Bhubaneswar, IND; 3 Neurological Surgery, National Institute of Nursing Education, Post Graduate Institute of Medical Education & Research, Chandigarh, IND

**Keywords:** covid vaccine, willingness, acceptance, unwillingness, hesitancy

## Abstract

Objective

To assess willingness for the coronavirus disease 2019 (COVID-19) vaccine and identify the factors attributing to the willingness.

Design

A cross-sectional study was conducted, adopting an exponential, non-discriminative snowball sampling technique. The questionnaire collected the socio-demographic profile, history of COVID-19 infection, presence of co-morbidities (diabetes mellitus, hypertension, chronic obstructive pulmonary disease (COPD), asthma, cancer), willingness, and preference of vaccine among participants. An online platform (Google Forms) was used to collect data from all over India. A total of 2032 Indian adults aged above 18 years were included in the study.

Results

Around 1598 (78.6%) expressed willingness to receive the COVID vaccine, and among the healthcare providers (HCPs), 579 (80.3%) were willing for COVID vaccination. Factors like the belief that the vaccine is necessary (aOR=1.68, 95% CI =1.34 to 2.11), respondents having no history of COVID infection (aOR=0.71, 95% CI: 0.52 to 0.97), having trust in the government (aOR=6.09, CI: 4.59 to 7.98), people who felt the cost of the vaccine didn’t matter (aOR=4.92, CI: 3.80 to 6.37), and respondents with no perceived risk of COVID infection (aOR=0.63; CI: 0.47 to 0.83) were more associated with willingness for COVID vaccination.

Conclusions

An effective vaccine should be well-received by the public. The responsibility lies with the government, health authorities, and manufacturers to take appropriate steps to dispel rumors in order to ensure people’s understanding and acceptance.

## Introduction

Introduction

Ever since the emergence of coronavirus disease 2019 (COVID-19) [1], the whole world has been looking towards scientists, researchers, and pharmaceutical companies to develop an effective vaccine against COVID-19. Globally, as of May 18, 2021, 15 vaccines have been approved by the health authorities; whereas, another 104 vaccines are at various phases of development [2]. Among the 15 vaccines, the Indian Government has approved three vaccines, namely ‘Covishield,’ ‘Covaxin,’ and ‘Sputnik V,’ which are being used for vaccination against COVID-19 among their citizens [3].

Since many decenniums under the universal immunization program (UIP), India has a splendid track record of running successful vaccination programs against numerous deadly infectious diseases [4-5]. Undoubtedly, gaining public trust and confidence is paramount for any vaccination program to be carried out extensively [6-7]. Owing to the coronavirus’s noxious potentiality, vaccines were developed, tested, and mandated to be used within a short period [8]. Speculations are high among the public regarding the safety and efficacy of such fast-track vaccines. With widespread cultural and regional diversity among the various states, India harbors a population of 1.38 billion people [9]. Amidst the fear of the COVID-19 pandemic and numerous reports of side-effects from newly developed COVID-19 vaccines [10-12], there is heightened apprehension and dilemma among the public to accept or reject the vaccination drive in the Nation. One of the significant hurdles against the acceptance of the vaccination drive is hesitancy, which could be aggravated by being ignorant, misinformation, culture, customs, beliefs, mistrust in authority, and fear of the vaccine’s adverse effects. In numerous countries, the vaccination drive against swine flu in 2009 wasn’t adequately accepted by the public and health professionals, owing to inadequate knowledge about the vaccine, worries about the unforeseen effects of vaccines, and the feeling that the vaccine is not necessary for adults [13-16].

The authors conducted an online public survey to estimate the public’s willingness to accept the COVID-19 vaccine and identify factors determining their acceptance of the vaccine. Similar studies were conducted in China, Indonesia, Saudi Arabia, and the United States that estimated the potential acceptance of the COVID-19 vaccine among their citizens [17-21]. All these studies were conducted well before the vaccine’s rollout, whereas the current study is conducted after the government’s official approval of the vaccine. Hence, the researchers assume that these findings would be the most accurate estimation of willingness and help identify specific factors behind their decision.

## Materials and methods

Study population and setting

**Figure 1:**
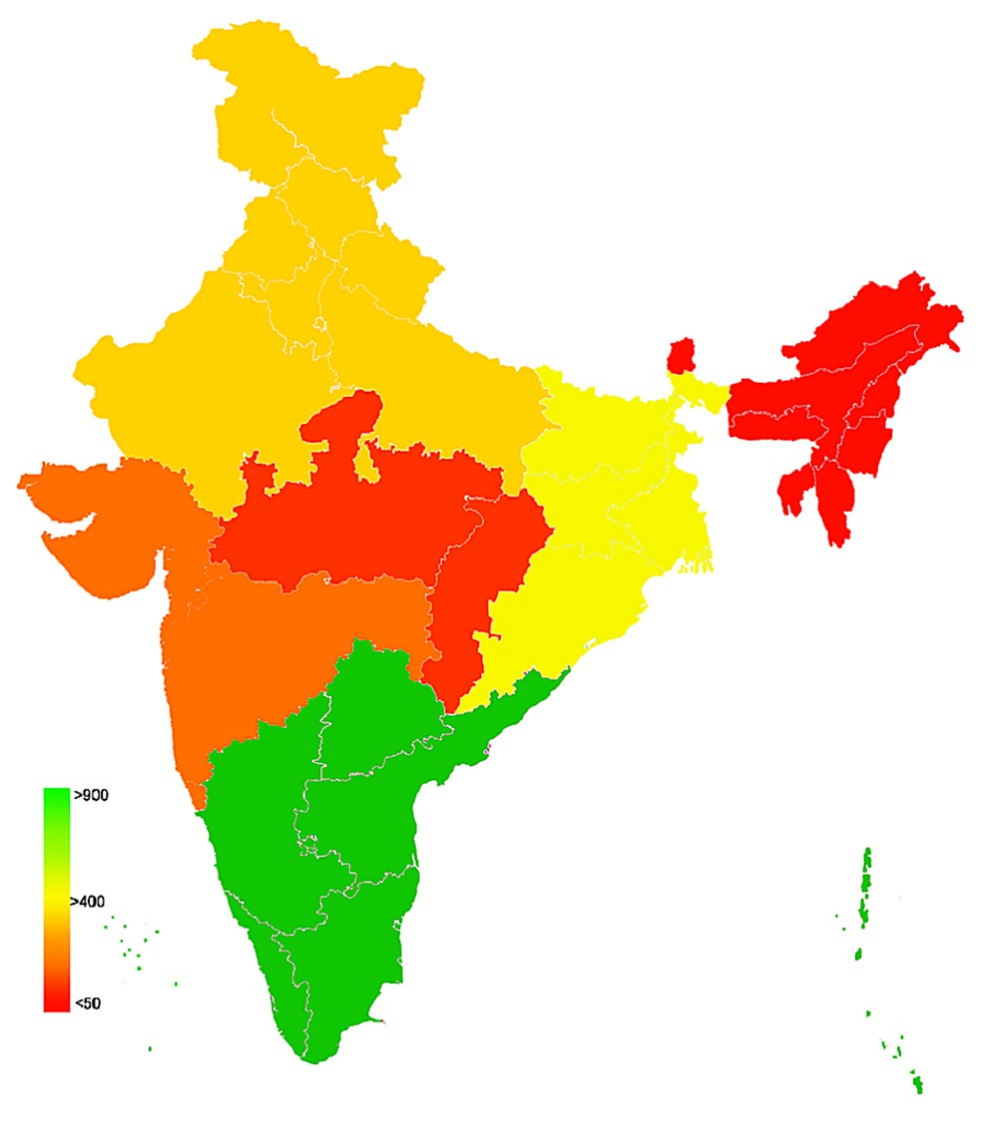
Geographical plot representing the zone-wise distribution of study participants

An online cross-sectional survey design was employed to assess the willingness for COVID-19 vaccination and identify the factors attributing to the willingness for COVID vaccination. The study participants comprised Indian citizens, aged 18 years or above, and those who could read and understand English. Participants’ recruitment was carried out from all the 28 states and eight union territories of India (Figure 1) [22].

Data collection tool

The researchers developed the questionnaires after reviewing relevant literature on vaccination programs during numerous past pandemics. Questionnaires comprised socio-demographic profile (age, gender, occupation, education, religion, place of residence, marital status, and monthly income), variables like participant’s history of a COVID-19 infection, history of COVID-19 infection among family members, presence of chronic co-morbidities (diabetes mellitus, hypertension, chronic obstructive pulmonary disease (COPD), asthma, cancer), and willingness, as well as the preference of vaccine (Indian, foreign, or any vaccine). A tool was constructed comprising 10 questions, five assessing reasons for willingness and the remaining five assessing reasons for unwillingness. The constructed questionnaire was pre-tested and validated by the experts. The average time required to complete the survey was 3-5 minutes.

Data collection procedure

Ethical approval was obtained from the Institution Ethics Committee of All India Institute of Medical Sciences, Bhubaneswar, vide Ref. Number T/IM-NF/Nursing/20/178. An exponential, non-discriminative snowball sampling technique was used to recruit the study participants. Data collection was done using an online questionnaire prepared on the Google platform (Google Forms). The link for participating in the study was disseminated to the public through social media platforms, namely, Gmail, Facebook, WhatsApp, and Telegram. The Google Forms' first page was provided with a detailed participant information sheet and consent agreement option. Those who consented were permitted to proceed further with the questionnaire. Data were collected from January 02 to January 14, 2021. The information filled in the questionnaire was updated in real-time on the Google Sheet, which was later exported for analysis.

Statistical analysis

Descriptive statistics were used to describe the demographic characteristics and outcome variables. The proportion of willingness for vaccines and their reasons were analyzed. Inferential statistic tests, namely, chi-square, odd’s ratio, and logistic regression were performed to determine the association of willingness for COVID-19 vaccine with variables like income, the status of COVID infection, perceived risk of COVID infection, trust in authority, cost of the vaccine, necessity of vaccination, and safety of the vaccine.

Chi-square was used to identify the variable having a significant association with willingness for the COVID-19 vaccine, which was further analyzed with odd’s ratio and logistic regression. All the tests were two-tailed, with a significance level set at p <0.05. Data were analyzed using Statistical Package for Social Sciences (SPSS) software version 17.0 (SPSS Inc., Chicago, Illinois).

## Results

Participant characteristics

Out of 2079 respondents, 2032 were considered for analysis after removing missing data and incomplete responses. The participants were recruited from all 28 states and eight Union territories (Figure 1), representing the six administrative zones (Northern, North-Eastern, Eastern, Western, Central, and Southern). The majority (959; 47.2%) of the respondents were from Southern states, and the minority (49; 2.4%) were from North-Eastern states. The mean age of the participants was 28.54+10.89 years and ranged from 18 to 94 years. About 1270 (62.5%) of participants were females, 1255 (61.8%) were unmarried, 1855 (90.3%) were holding bachelor degree, 722 (35.53%) were health care providers (HCP), 255 (12.5%) had a history of COVID infection, 361 (17.8%) had a history of family members being COVID positive, 188 (9.3%) had a chronic illness, and nearly half (991; 48.8%) were from the low-income category (Table 1).

**Table 1:** Demographic variables of the study participants

Variables	Frequency	Percentage (%)
Age (in years)		
18-24	1009	49.65
25-55	949	46.70
>55	74	3.50
Gender		
Male	762	37.5
Female	1270	62.5
Marital status		
Single	1255	61.8
Married	754	37.1
Divorced/separated/widow/widower	23	1.1
Education status		
Matriculation and below	177	8.7
Bachelors degree	1205	58.3
Post-graduation and above	650	32
Occupation		
Health care provider	722	35.53
Others	1310	64.47
Income (per month)		
Low (<10000 INR/<137 USD)	991	48.8
Fair (10001 INR to 30000/138-410 USD)	574	28.2
High (>50000 INR/411-684 USD)	467	23
Residence		
Rural	557	27.4
Semi-urban	487	24
Urban	988	48.6
Religion		
Hindu	1390	68.40
Christian	461	22.69
Muslim	141	6.94
Others	40	1.97
History of COVID infection (Self)	255	12.5
History of COVID infection (Family members)	361	17.8
Presence of chronic illness	188	9.3

Willingness and preference for COVID-19 vaccine

**Figure 2:**
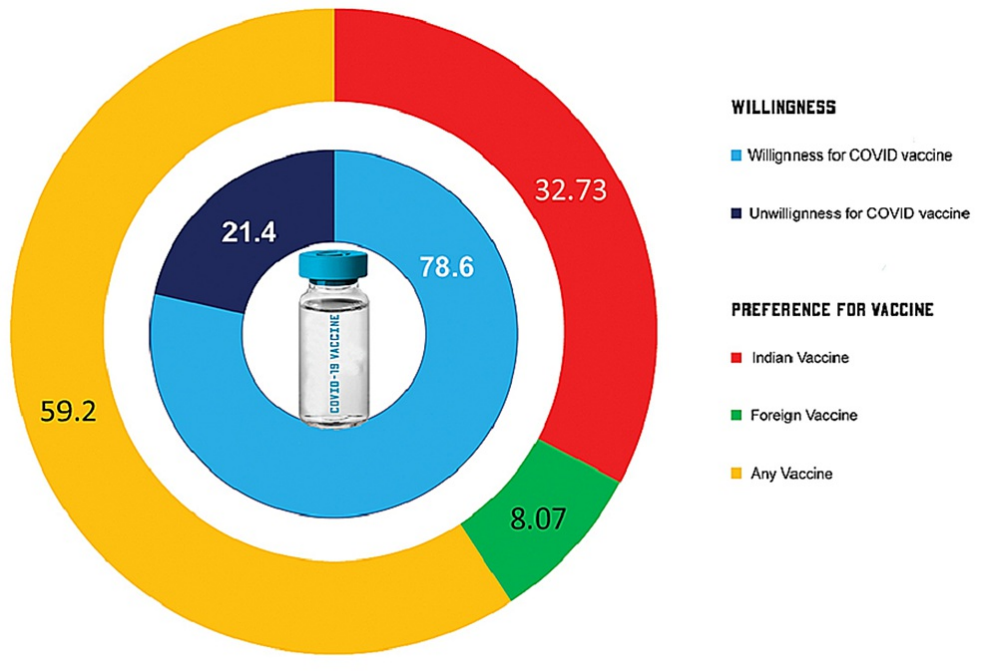
Willingness and preference for vaccine among Indians

Of the total respondents, 1598 (78.6%) expressed willingness to receive the COVID-19 vaccine (Figure 2). Among the HCPs, 579 (80.3%) were willing for the COVID-19 vaccine, whereas 185 (72.9%) of the respondents had a history of COVID infection and 271 (75.3%) of the respondents who had a history of family members being COVID positive were willing for COVID-19 vaccination. Most of the respondents with co-morbidities (140; 74.5%) were willing for the COVID-19 vaccine. The highest proportion of willingness was seen among the Western zone (102; 80.3%), followed by the Northern (286; 79.9%), Southern (766; 79.8%), and Eastern (347; 76.1%) zones of India. The lowest proportion of willingness was recorded from the North-Eastern (36; 75%) and Central (61; 73.5%) zones of India. Among the willing respondents, 523 (32.73%) preferred vaccines developed by Indian pharmaceutical institutes, and 129 (8.07%) preferred foreign vaccines. The remaining 946 (59.20%) preferred either of the two vaccines (Figure 2).

Reasons for willingness and unwillingness for COVID-19 vaccination

**Figure 3:**
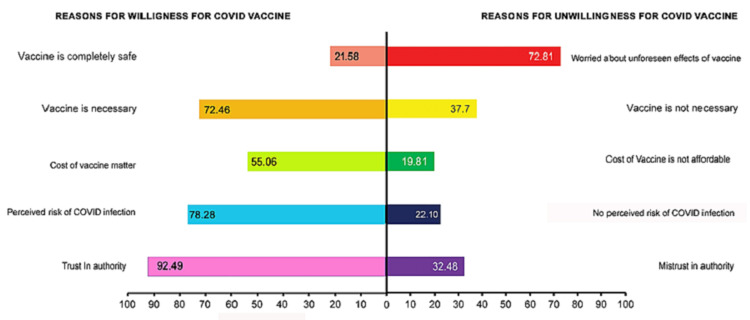
Reasons for willingness and unwillingness for a COVID-19 vaccine

The current study revealed that the major reason for willingness to receive the COVID-19 vaccine was trust in authority (92.49%), followed by the perceived risk of COVID infection (78.28%), and a belief that vaccination is necessary for the prevention of COVID (72.46%) (Figure 3). Among 2032 respondents, 21.4% (434) were unwilling for a COVID-19 vaccine, and the major reasons for unwillingness were attributed to ‘worried about unforeseen effects of vaccine’ (72.81%), followed by the belief that the vaccine is not necessary (37.7%) and mistrust in authority (32.48%).

Determinants of willingness for COVID-19 vaccination

Findings reveal that respondents with a low income were 1.48 times more willing for vaccination than fair income groups (aOR=1.48, 95% CI: 1.13 to 1.94) (Table 2). The respondents who had no history of COVID infection were 29% more willing than those with a history of COVID infection (aOR= 0.71, 95% CI: 0.52 to 0.97). Participants who trusted in authority were 6.09 times more willing to receive the vaccine than respondents who don’t trust authority (aOR=6.09,95% CI: 4.59 to 7.98). Participants for whom the cost of vaccine mattered were 4.92 times more willing for vaccination than those for whom the cost of the vaccine didn’t matter (aOR= 4.92, 95% CI =3.806.37). The respondents who believed that a vaccine is necessary were 1.68 times more willing for vaccination than those who felt the vaccine is not necessary (aOR=1.68, 95% CI =1.34 to 2.11). On the contrary, respondents with no perceived risk of COVID infection were 27% more willing to vaccinate than participants with perceived risk (aOR=0.63; 95% CI: 0.47 to 0.83). Lastly, respondents who felt the vaccine is not completely safe were 29% more willing for vaccination than those who felt the vaccine is completely safe (aOR=0.71, 95% CI: 0.55 to 0.90).

**Table 2:** Univariate regression to identify the determinant of willingness for COVID vaccination OR: odds ratio, †aOR: adjusted odds ratio (adjusted for age, gender, occupation, zone, place of residence, religion, and marital status) *p<0.01, **p<0.001, #OR and aOR were calculated using binary logistic regression; Ref: reference category

Sl no	Variable (reference variable)	OR (95% CI)	aOR^†^ (95%CI)
1	Income		
	Fair (Ref)		
	High	0.89 (0.67 to 1.18)	0.91(0.68 to 1.22)
	Low	1.45* (1.12 to 1.86)	1.48*(1.13 to 1.94)
2	History of COVID infection		
	No (Ref)		
	Yes	0.69*(0.51 to 0.93)	0.71*(0.52 to 0.97)
3.	Perceived risk of COVID infection		
	No (Ref)		
	Yes	0.63* (0.47 to 0.83)	0.63* (0.47 to 0.83)
4	Trust in authority		
	No (Ref)		
	Yes	6.10** (4.64 to 8.03)	6.09** (4.59 to 7.98)
5	Cost of vaccine matters		
	No (Ref)		
	Yes	4.93** (3.82 to 6.37)	4.92**(3.80 to 6.37)
6	The vaccine is necessary for the prevention of COVID-19		
	No (Ref)		
	Yes	1.68** (1.35 to 2.10)	1.68** (1.34 to 2.11)
7	COVID-19 vaccine is completely safe		
	No (Ref)		
	Yes	0.74* (0.58 to 0.94)	0.71* (0.55 to 0.90)
OR: odds ratio,^ †^aOR: adjusted odds ratio (adjusted for age, gender, occupation, zone, place of residence, religion, and marital status ) *p<0.01, **p<0.001, ^#^OR & aOR was calculated using binary logistic regression,			

## Discussion

Since Edward Jenner’s days, vaccines have been the foundation for preventing viral infections [23]. The world has been fighting COVID-19 since December 2019, and to date, a definitive treatment is not available. Hence, a vaccine has remained the only solution to control the pandemic. In a democratic country like India, the vaccine program’s success depends on the way people perceive and accept the initiative, which may vary with time, place, religion, and culture.

In the current study, nearly half (49.65%) of participants were within the age group of 18-24 years, reflecting the young generation’s - who actively express their views and concerns about socially relevant issues - presence on social media platforms. Such young people’s decision strongly reflects the community’s perception, judgment, and attitude.

The proportion of participants’ willingness to receive the COVID-19 vaccine revealed in the present study was similar to other studies worldwide. Among the reported studies, the highest proportion of willingness was observed from Indonesia (93.3%) [20], and the lowest proportion was reported from Russia [24]. Our study has shown a moderate level of acceptance, which can be explained in two ways. First, India had more than 10 million COVID cases [25], and the public might have wanted to feel safer. Second, the willingness for the vaccine may have been dented, probably owing to the fact that cases were on a declining trend in India during the time of data collection (January 2021) and active cases were around 0.2 million cases [26], creating an intuition that vaccine is no longer necessary.

In this study, HCPs had a higher proportion of willingness for the COVID-19 vaccine, which is similar to the study reported from China, which depicted that 76.4% of HCPs were willing for COVID vaccination [27]. Like in other nations, HCPs are the first recipients of the COVID-19 vaccine. This can be justified because HCPs are the frontline warriors in the war against this pandemic. Moreover, the HCPs have much better knowledge about the disease and vaccination than the general public [27].

The major reasons for unwillingness to receive the COVID-19 vaccine was "worried about unforeseen effects of the vaccine" and "vaccine is not necessary" (believing in natural immunity) in combating the infection. A similar finding from the United Kingdom reflected that worries about unforeseen effects (RRR=4.91; CI: 3.76 to 6.42) and a strong preference for natural immunity (RRR=2.51; 95% CI: 1.78 to 3.53) were the contributors for unwillingness towards vaccination [28]. This may be attributed to the fact that vaccines were fast-tracked, and some of them were rolled out for emergency usage even without publishing the results of phase III trials, creating lacunae among the public [3]. Occurrence and media reports of events like paralysis, breathing difficulty, and death as an adverse effect of vaccines can further dent the vaccination’s reputation and credibility [10-12].

The logistic regression results revealed that “trust in authority" is one of the most influential determinants for willingness for the COVID-19 vaccine. The findings are congruent with studies conducted in Saudi Arabia [18] (aOR=3.05; 95% CI=1.13 to 4.92) and globally (aOR=1.67; 95% CI=1.54 to 1.80) [24]. In a democratic country like India, trust in authority is of utmost importance for any population-based health activity to be successful, and this is reiterated in our study. On the contrary, in the current study, the mistrust in authority was one of the major reasons for unwillingness for the COVID-19 vaccine. This is supported by the study reports from the United Kingdom, where concern about commercial profit (RRR=1.73; 95%; CI=1.34-2.24) was predominant for unwillingness [28].

In the current study, participants with no perceived risk of COVID infection were more willing for the COVID-19 vaccine, which is contrary to the finding from Saudi Arabia (aOR=2.13; 95% CI: 1.35 to 3.85) [18] and Indonesia (aOR=2.21; 95% CI: 1.07 to 4.59) [20]. This could be since similar studies were conducted when COVID cases were escalating. The current study was conducted when cases in India were declining [26], which ceases its significance.

Table 2 reveals that the cost of the vaccine matters for the willingness for the COVID-19 vaccine. This finding contradicts a study from China [17], which reported that price is not an important factor for willingness for the vaccine (OR=0.75; CI: 0.61 to 0.93). This can be justified due to the low-income category respondents in the present study. Moreover, India is a developing country where the price of the vaccine impacts the respondents.

Studies concerning the public’s acceptance and attitude towards the COVID-19 vaccine have been done in other parts of the world during the initial phases of the COVID outbreak. But studies after vaccine approval are very few. Hence, the current study is significant to identify the determinants contributing towards willingness for COVID-19 vaccination among Indians.

Limitations

This study has some limitations; it was an online survey with a relatively small sample size compared to India’s large population. Uncertainty about the reliability of the questionnaire’s source, fear of online fraudulence, etc., could have attributed to the lower response rate. Despite the researchers providing detailed participant information sheets in the questionnaire, these factors might have prevented many from responding with actual information. Hence, information bias is probable. However, such geographically broadband cross-sectional studies are possible only through online mode. To overcome the above bias, we have tried to receive responses from all corners of India.

Furthermore, disproportionate response for various states, education status, occupation, and past experiences might have created a bias in generalizing the findings. Also, people who have access to and an understanding of answering an online questionnaire might have adequate knowledge and might well read about the vaccines. But their view may not represent the views of a larger community of India who lack the skill or facility for online response.

Strengths and implications

The study reveals that willingness or unwillingness is not due to a single factor; instead, a complex interaction of many beliefs makes it up. The government and health agencies have to tackle these issues by providing clear-cut information through various forms of communication and taking actions that will develop trust towards a government, which can be reflected in the COVID vaccination drive.

## Conclusions

The government has to work on measures to improve the general public’s knowledge of and attitude regarding COVID vaccination. Efforts have to be directed to all, irrespective of caste, religion, color, customs, and language. Communication has to be direct and straightforward and prevent misinformation from spreading from unknown sources. All these actions will improve trust in the government/authority and reduce worries about the unforeseen effects of the vaccine. Lastly, the acceptance of this vaccine will help humankind overcome the inevitable enemy, the ‘COVID’ pandemic.
